# SNPs or RNA modifications? Concerns on mutation-based evolutionary studies of SARS-CoV-2

**DOI:** 10.1371/journal.pone.0238490

**Published:** 2020-08-28

**Authors:** Yue Li, Xinai Yang, Na Wang, Haiyan Wang, Bin Yin, Xiaoping Yang, Wenqing Jiang

**Affiliations:** Department of Respiratory Diseases, Qingdao Haici Hospital, Qingdao, China; John Curtin School of Medical Research, AUSTRALIA

## Abstract

SARS-CoV-2 is still rampaging throughout the world while the many evolutionary studies on it are simultaneously springing up. Researchers have simply utilized the public RNA-seq data to find out the so-called SNPs in the virus genome. The evolutionary analyses were largely based on these mutations. Here, we claim that we reliably detected A-to-G RNA modifications in the RNA-seq data of SARS-CoV-2 with high signal to noise ratios, presumably caused by the host’s deamination enzymes. Intriguingly, since SARS-CoV-2 is an RNA virus, it is technically impossible to distinguish SNPs and RNA modifications from the RNA-seq data alone without solid evidence, making it difficult to tell the evolutionary patterns behind the mutation spectrum. Researchers should clarify their biological significance before they automatically regard the mutations as SNPs or RNA modifications. This is not a problem for DNA organisms but should be seriously considered when we are investigating the RNA viruses.

## Introduction

The outbreak of SARS-CoV-2 (Severe Acute Respiratory Syndrome Coronavirus 2) in the beginning of year 2020 has caused severe damage to China especially the Hubei province [1–3]. Recently, most countries in the world are consecutively being hit by this virus. There is urgent need to understand the origin and evolution of SARS-CoV-2 and related coronaviruses [4].

Papers on the evolutionary patterns of SARS-CoV-2 have emerged as rapidly as the outbreak of virus. Several studies downloaded the publically available RNA-seq data and performed “SNP calling” or sequence alignment. The SNP distribution or frequency spectrum acts as a super informative inference of selection patterns. The recent study has discussed topics on the origin and continuing evolution of SARS-CoV-2. Although the methodology of some studies was challenged by other researchers [5], their naïve attempt to call SNP from the SARS-CoV-2 RNA-seq data is unchallenged.

To better illustrate the pipeline of SNP calling, let us take the human genome for instance. The SNP calling process is accomplished by mapping the DNA-seq reads (of a sample) to the human reference genome, and the reliably detected mismatch sites should be SNPs. If the RNA-seq data of the same sample is available, one could find the same nucleotide changes from the reference genome to the RNA-seq reads, indicating that the mutation takes place at DNA level. However, one could not determine the direction of the mutations without an outgroup species. In contrast, if a variation site is only found in the RNA-seq reads but not the DNA-seq reads, then this is possibly an RNA modification site. For example, the vertebrate adenosine deaminase would change adenosines to inosines [6], which would be interpreted as guanosines in the sequencing data. Thus, without the support of DNA-seq data, the A-to-G variations between RNA and reference genome are presumably caused by the deamination enzyme. Unlike the unknown direction of DNA mutations, the direction of A-to-G deamination is very clear even without an outgroup because it is the adenosine to inosine change at RNA level.

Researchers should note that SARS-CoV-2 is a positive strand RNA virus. The so-called reference genome is actually the RNA sequence. Without a DNA template, the mismatches found between the reference and the RNA-seq reads could either be a “SNP” or an RNA modification site. It is futile to try any filtering cutoffs on these variation sites because the SNPs and RNA modification sites are technically indistinguishable. Application of cutoffs only makes the variants more reliable but does not help distinguish whether the variants are SNPs or RNA modifications. Even when multiple outgroup species are available, the reference sequence (RNA) of the outgroup viruses may also undergo the same RNA modification process (by host cells), making it difficult to define the ancestral state and the direction of mutations.

**Fig 1:**
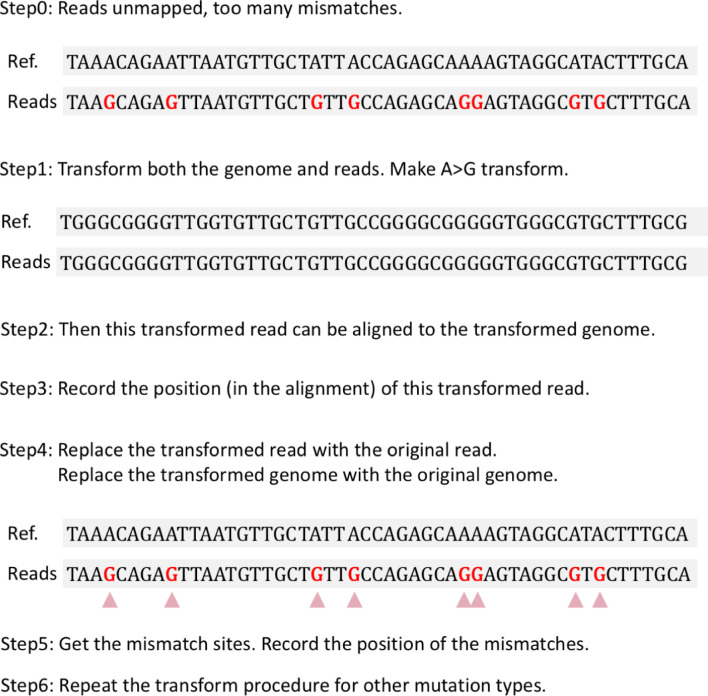
The transform strategy of mapping the reads with multiple mismatches.

In this study, using a well-established mutation finding pipeline (see **Fig 1** and Materials and methods), we found prevalent A-to-G RNA modifications in the RNA-seq data of SARS-CoV-2. Questions come that the natural mutations should be randomly caused by RNA replication errors and should not have a preference on A-to-G mutations. Therefore, the A-to-G variations are likely to be caused by the RNA deamination system while other non-A-to-G variations might be the so-called SNPs produced by replication errors. The RNA replication errors and RNA modification system should have completely different mutation rates and evolutionary patterns. Thus, mixing all the variation sites does not make sense and could not accurately reflect the evolution history. It is peculiar to study the origin and evolution of SARS-CoV-2 by investigating all the mixed variation types. The simple aim of this current study is to reveal that the mutation profile of SARS-CoV-2 is indeed skewed. We were unable to provide a solution to cancel the bias but we intend to remind the broad researchers to notice this bias and avoid obtaining an inaccurate conclusion.

## Materials and methods

### Data collection

We downloaded the novel coronavirus SARS-CoV-2 genome from the NCBI website (https://www.ncbi.nlm.nih.gov/genome/). The coding sequences were extracted according to the genome annotation. The RNA-seq data were retrieved from NCBI under accession numbers SRR10903401, SRR10903402, SRR11059942, and SRR11059945.

### RNA-seq analyses

We mapped the RNA-seq reads to the CDS of SARS-CoV-2 using BWA mem [7] with default parameters but with a little modification (**Fig 1**). Reads with too many mismatches could not be aligned to the reference genome. However, the multiple clustered mismatches could be RNA modification events. To retrieve more RNA modification sites in clusters like this, we manually transformed the reference sequence and the RNA-seq reads [8]. We performed the transformation for all of the twelve mismatch types (with one mismatch type each time). If any transformed reads could be mapped to the transformed genome, then we record the positions of the reads on the genome (termed coordinates), and the transformed reads and genome were replaced with the original reads and the unmodified genome. The mismatch sites between the original reads and genome were extracted from the alignment. By this way, an unmapped read (due to too many mismatches) could be successfully mapped to the genome. We made two versions here, one version is the variation sites without additional filters, another is the mutation sites under the criteria of mapping quality > 25 and base quality > 35. The “transformation followed by re-mapping” workflow is a well-acknowledged pipeline to detect the RNA modification sites omitted by traditional mapping procedures [8, 9]. However, this protocol did not consider mapping quality and base quality, so that we need to reproduce this pipeline with additional filters. We also emphasize that this pipeline only deals with the reads that could not be mapped by normal procedures [10].

Codes of mapping and variant calling:

Mapping:

bwa index reference.fasta

bwa mem reference.fasta sample.fastq > sample.sam

Variant calling:

samtools mpileup–q 25 –Q 35 sampleX.sam > sampleX.vcf

### Statistical analyses

We used the R language to perform the statistical analyses and graphic work. We also used EXCEL to plot some figures when necessary.

## Results

### Variation sites identified by normal mapping pipeline

A recent study [10] dealt with similar issues by using the same sets of data. As we have said, the mutation sites found by traditional variant calling pipeline only represent a minority of all possible RNA modification sites in the transcriptome, and usually would not produce a strikingly high percentage of A>G mutation. In the next sections, we would no longer discuss these sets of variation sites. We would use the transform strategy as introduced in the Materials and methods (**Fig 1**) and look at the clustered modification events across the transcriptome.

### The prevalent A-to-G variations across SARS-CoV-2 genes

**Fig 2:**
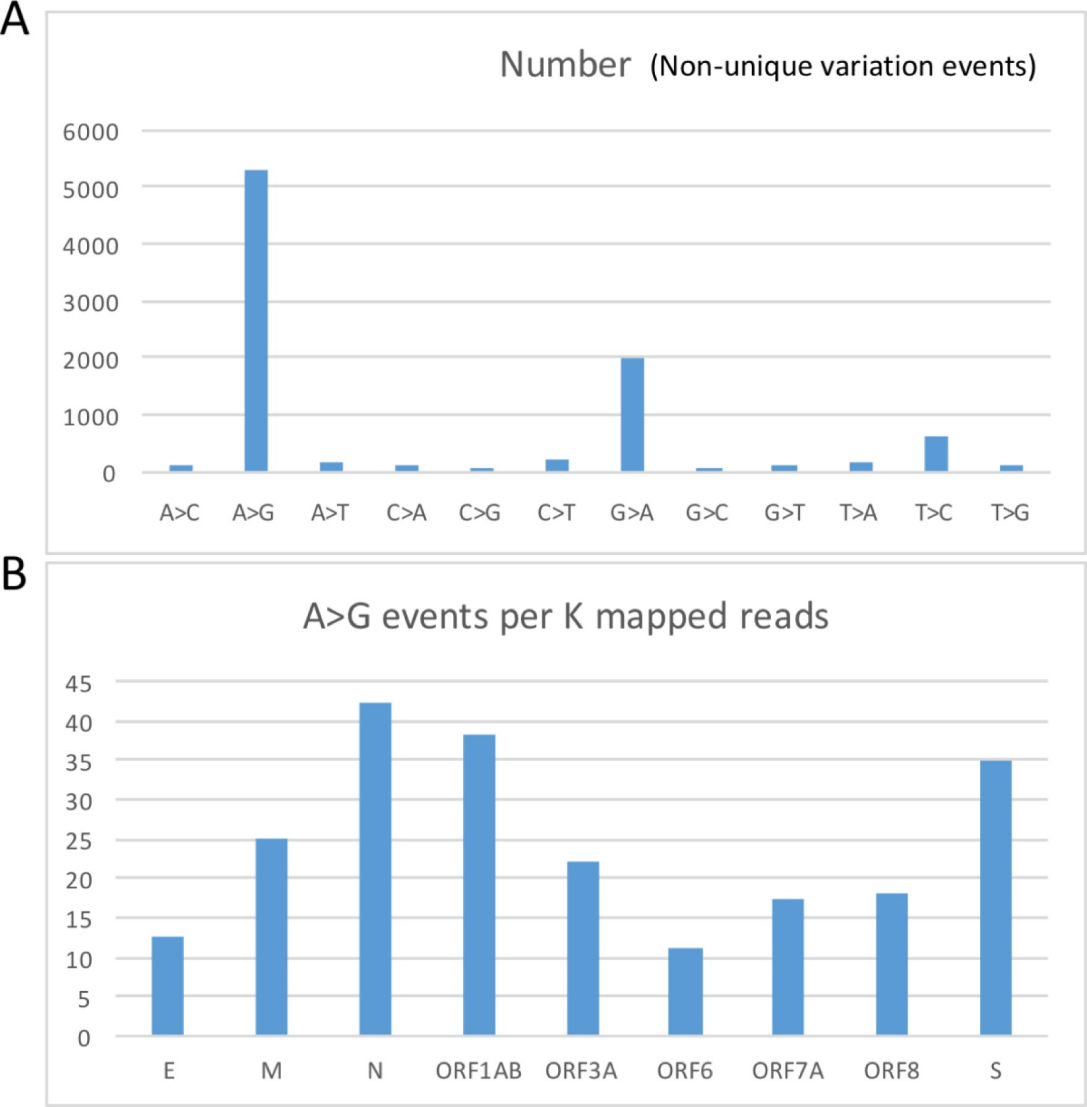
The mismatch profile of a set of RNA-seq data from SARS-CoV-2. **(A)** Numbers of mismatch events (not unique sites). **(B)** The normalized number of A-to-G mismatches per gene. There are totally 11 non-redundant genes in the SARS-CoV-2 sequence, and the A-to-G alterations are found in 9 of those genes.

We downloaded the reference and a set of RNA-seq data of SARS-CoV-2. We mapped the RNA-seq reads to the reference sequence with a well-established pipeline (see Materials and methods for details). The numbers of (non-unique) mismatch events profiled (**Fig 2A**). There are 5310 (59.1%) A-to-G mismatches and 2015 (22.4%) G-to-A mismatches, and the ten other types of mismatches composed only 18.5% of the totally 8989 mismatches (**Fig 2A**). The most prevalent A-to-G mismatches could be interpreted as the adenosine-to-inosine deamination conferred by the host cells. For the second-most prevalent G-to-A mismatches, it is possible that the reference sequence (RNA) of SARS-CoV-2 itself suffered from A-to-G deamination by the host cells, and the nucleotide in the RNA-seq is the unmodified version (adenosine). The third-most abundant T-to-C and C-to-T mismatches might represent the cytosine-to-uridine deamination system in the host cells.

**Fig 3:**
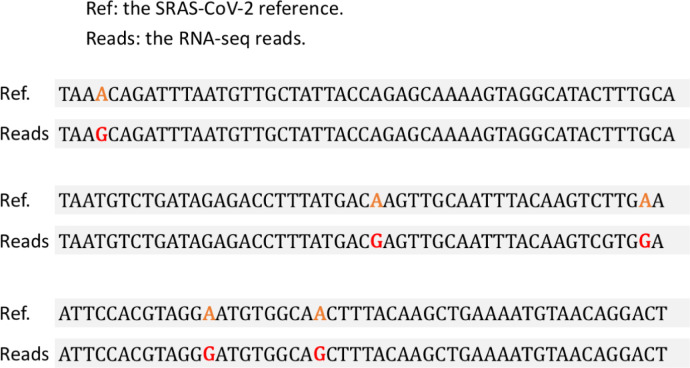
An example of an alignment between an RNA-seq read and the reference sequence of SARS-CoV-2. The five A-to-G mismatch sites are colored in the Fig.

We treated the A-to-G as the A-to-G RNA modification sites in the virus sequences. We found that the density of A-to-G modification varied moderately across different genes (**Fig 2B**). To technically validate the reliability of the mismatch sites, we manually extracted a 150bp read and aligned it to the reference sequence (**Fig 3**). The A-to-G alterations are clearly presented in the alignment. Note that the terminology “error” could refer to mis-alignments or sequencing errors. The validation here is to check the accuracy of the mapping pipeline. The manually examined read only proved that the sequence alignment is reliable. The control for sequencing errors would be discussed in the following section.

### Robustly observed A-to-G variations under different criteria

**Fig 4:**
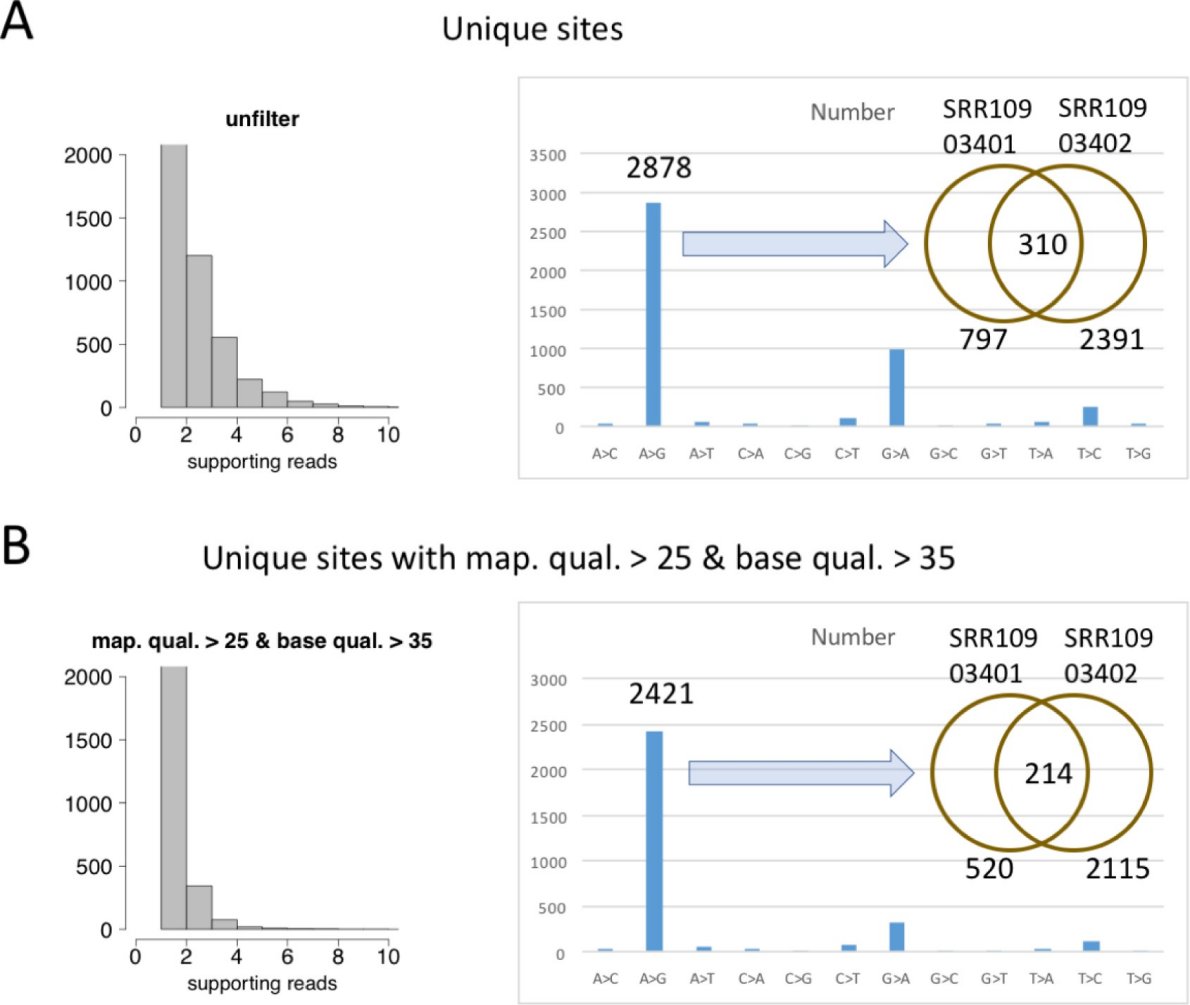
The numbers of unique mutation sites. (A) Distribution of reads count supporting each variation site and the numbers of different mutation types. This is the unfiltered results. (A) Distribution of reads count supporting each variation site and the numbers of different mutation types. This is the results with mapping quality > 25 and base quality > 35.

The nearly nine thousand (non-unique) variation events shown in the above section belong to 4604 unique variation sites. Most of the 4604 unique sites have less than 10 reads supporting the alternative allele (**Fig 4A**). There are 2878 (62.5%) unique A-to-G variation sites and 998 (21.7%) unique G-to-A variation sites (**Fig 4A**). The signal to noise ratio for A-to-G variations is 1.67. If combined with G-to-A variations, then the signal to noise ratio would be as high as 5.32. Among the 2878 unique A-to-G sites, 797 and 2391 sites were found in the two SRR samples, respectively, and 310 sites were overlapped.

Since these results came from the mapping and variant calling procedures without any filtering criteria, we think it is necessary to see whether the patterns are sensitive to any filtering parameters. We re-did the analysis by requiring mapping quality > 25 and base quality (controlling for sequencing errors) > 35. We found that the number of unique variation sites (3129) slightly declined but the majority (2421, 77.4%) of which are still A-to-G variations (**Fig 4B**). This result confirmed that the prevalent A-to-G variants would not be affected by any filtering cutoffs.

**Fig 5:**
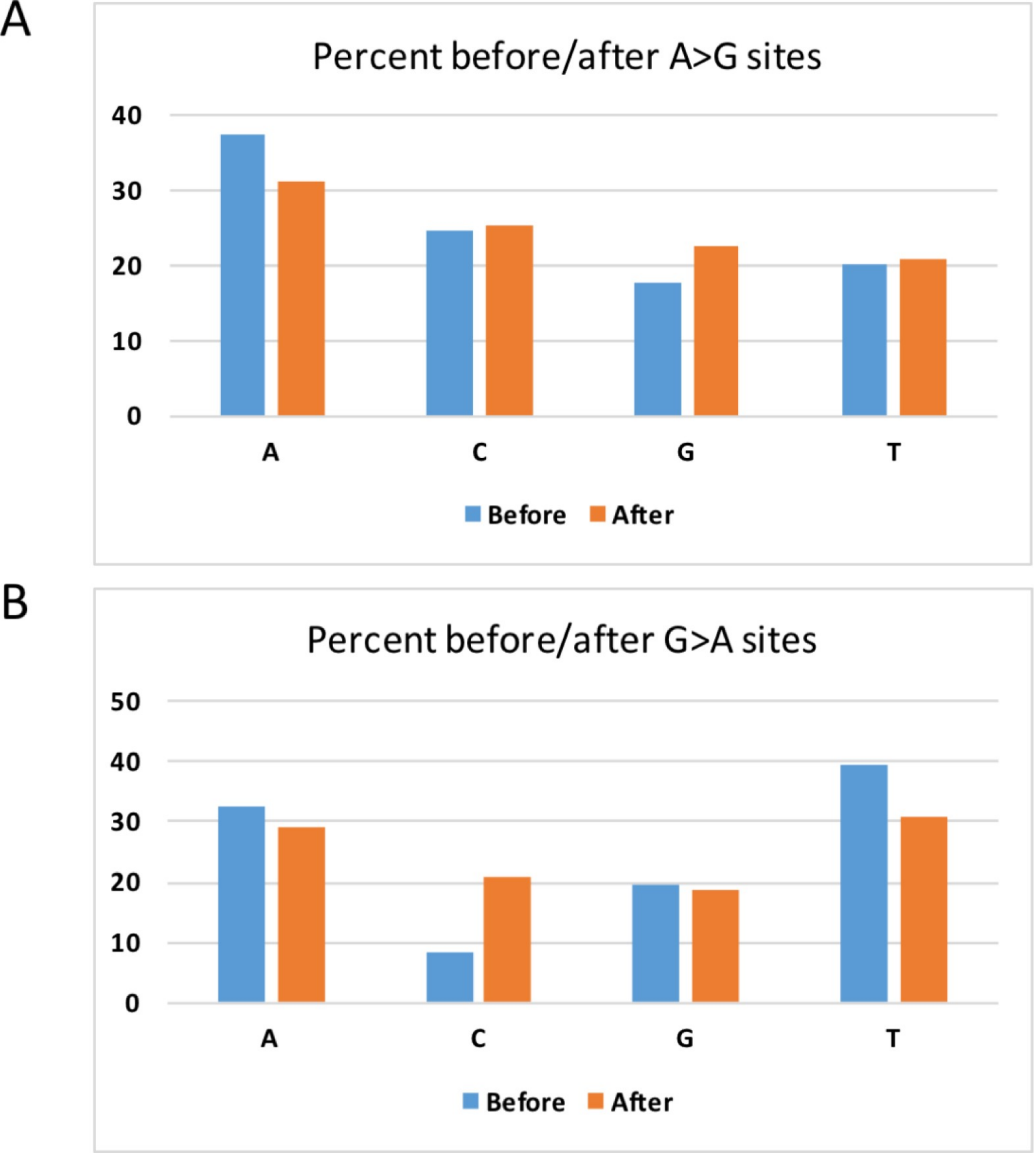
The base context of unique mutation sites. The percentage of A, C, G, and T is provided as bars. (A) Context of A-to-G sites. (B) Context G-to-A sites. "Before" means the five-prime nucleotide. "After" means the three-prime nucleotide.

We also wish to prove the reliability of the putative A-to-G modification sites from another angle. The base context of the A-to-G variation sites showed an obvious depletion of G upstream to the putative A-to-G modification sites (**Fig 5A**). Note that this depletion of G is statistically significant under Chi-square tests with the null hypothesis of equal numbers of A, C, G, and T (p-value < 1e-4). In contrast, the G-to-A variation sites did not have such a key validation feature (**Fig 5B**). This consolidated our assumption that these A-to-G variations are RNA modification sites.

## Discussion

The SNPs and RNA modification sites could bear completely different mutation rates and position biases, and also undergo different selection patterns and evolutionary trajectories. Analyzing the mixture of SNPs and RNA modification sites does not make sense. It does not reflect the evolutionary patterns of either SNPs or RNA modification. Unfortunately, these two mutation sources could not be separated from the RNA-seq data of RNA viruses. This uncertainty would confound any evolution studies involving sequence alignments. For example, presume the non-A-to-G mutation rate is N1 per generation and the A-to-G deamination rate is 10*N1 per generation. By mixing all mutations, one would obtain a mutation rate around 5*N1 per generation. This rate is neither the mutation rate (caused by replication error) nor the deamination rate.

In addition, the deamination process also obscures the inference of ancestral state. If it is confirmed that the SARS-CoV-2 has been transferred from patient No.1 to patient No.2, then one might consider the RNA-seq from patient No.1 should be the ancestral state. However, the viral RNAs in patient No.1 would undergo A-to-G modification by the hosts. The deamination enzyme only modifies a fraction of the total viral RNAs so that in patient No.1 there is still a mixture of A-version and G-version RNA reads. Technically, one could not know whether this is a polymorphic site in the virus population or it is modified by the host’s enzyme. This uncertainty makes it difficult to define the ancestral state.

There is a less important but unsolved question that we think the G-to-A variation sites could also be the A-to-G modification on RNA of the “reference genome”. However, from the base context of the G-to-A sites, they did not seem to be authentic A-to-G modification sites. This strange pattern remains an open question.

In summary, for many studies that claimed the optimization in variant calling pipeline, they only improved the accuracy of the alignments. Even an alignment is manually verified, we still do not know whether the A-G mismatches in the alignment should be SNPs or RNA modification sites. We appeal that this issue should be seriously discussed in the studies involving RNA viruses like SARS-CoV-2.

## Conclusions

The technically indistinguishable RNA modifications and SNPs of SARS-CoV-2 have complicated the situation where the researchers intend to reveal the evolutionary patterns behind the mutation spectrum. This is not a problem for DNA organisms but should be seriously considered when we are investigating the RNA viruses.
